# Emulating spin transport with nonlinear optics, from high-order skyrmions to the topological Hall effect

**DOI:** 10.1038/s41467-021-21250-z

**Published:** 2021-02-17

**Authors:** Aviv Karnieli, Shai Tsesses, Guy Bartal, Ady Arie

**Affiliations:** 1 School of Electrical Engineering, Fleischman Faculty of Engineering, Tel Aviv University, Tel Aviv, Israel; 2 Andrew and Erna Viterbi department of Electrical Engineering, Technion—Israel Institute of Technology, Haifa, Israel

**Keywords:** Nonlinear optics, Spintronics

## Abstract

Exploring material magnetization led to countless fundamental discoveries and applications, culminating in the field of spintronics. Recently, research effort in this field focused on magnetic skyrmions – topologically robust chiral magnetization textures, capable of storing information and routing spin currents via the topological Hall effect. In this article, we propose an optical system emulating any 2D spin transport phenomena with unprecedented controllability, by employing three-wave mixing in 3D nonlinear photonic crystals. Precise photonic crystal engineering, as well as active all-optical control, enable the realization of effective magnetization textures beyond the limits of thermodynamic stability in current materials. As a proof-of-concept, we theoretically design skyrmionic nonlinear photonic crystals with arbitrary topologies and propose an optical system exhibiting the topological Hall effect. Our work paves the way towards quantum spintronics simulations and novel optoelectronic applications inspired by spintronics, for both classical and quantum optical information processing.

## Abstract (web summary)

Control of effective magnetization textures like skyrmions is limited by the thermodynamic stability in current materials. Here, the authors propose a 3D nonlinear photonic crystal to emulate 2D spin transport phenomena with excellent controllability.

## Introduction

Exploring the physics of material magnetization has long been a focal point of both fundamental science and technological advances. The study of various magnetic phases such as spin ice^1^ and spin glass^2^ unveiled novel fundamental phenomena, e.g., magnetic monopoles^3^, while giant magneto-resistance^4^ and spin currents^5^ facilitated applications of magnetic information transfer and storage, giving birth to the field of spintronics^6^.

A recent focus in this field is on magnetic skyrmions^7,8^: 3D topological defects in 2D magnetization textures, which are robust to disorder and can be driven in an energy-efficient manner^9^, making them excellent candidates for memory applications and information processing^10^. Skyrmions can also be applied to control spin transport through the topological Hall effect^11–13^: the deflection of a spin-1/2 particle due to its interaction with a topologically nontrivial magnetization.

Although conducting spin transport experiments is readily achievable, performing them under arbitrary magnetization conditions is difficult, since both natural and artificial magnetic materials are restricted to thermodynamically stable phases^14,15^. Likewise, exact control over spin currents often requires cryogenic temperatures and external control fields, which may influence the system Hamiltonian^16,17^. As such, utilizing more controllable physical systems to implement the required interaction may be of great importance, both for exploring the system dynamics and for discovering new effects and applications.

For example, the quantum Hall effect^18^ was successfully simulated using systems the likes of cold neutral atoms^19,20^ and electromagnetic waves^21,22^ by virtue of artificial gauge fields^23–25^. In optics, wherein fabrication capabilities allow a high degree of controllability and straightforward measurement, these experimental analogies ultimately created the field of topological photonics^26^, enabling many exciting applications, including topologically protected lasing^27,28^.

Motivated by the recent discovery of skyrmions in optics^29–31^, and the ability of nonlinear optical processes to effectively define a spin-1/2 system^32–34^, we propose a method to emulate any 2D spin transport phenomenon with light, using 3D nonlinear photonic crystals (NLPCs)^35–38^. As a proof-of-concept, we analytically and numerically present an emulation of the topological Hall effect by engineering effective skyrmion textures for light. The effective magnetization in our proposed system is highly tunable, such that high-order skyrmion textures and domain wall fine structures, otherwise unstable in magnetic materials, may be created to probe spin transport dynamics. We also suggest methods for active, all-optical control over the effective magnetization in the NLPC, illustrating the potential of our approach to support the development of new optical and quantum optical devices inspired by spintronics. Our formalism applies to any 2D magnetization landscape and can even be extended to simulate spin transport through time-dependent magnetizations, such as melting domains^39^ and spin waves^40^. Employing the high availability of single-photon sources, our formalism may allow the quantum simulation of single-particle phenomena such as Anderson localization^41^ or quantum random walk^42^ of spin-carrying particles as well as transport phenomena with entangled spins^43^, which can be simulated using frequency-entangled multiphoton states.

## Results

### Spin transport emulation through nonlinear optics

**Fig. 1:**
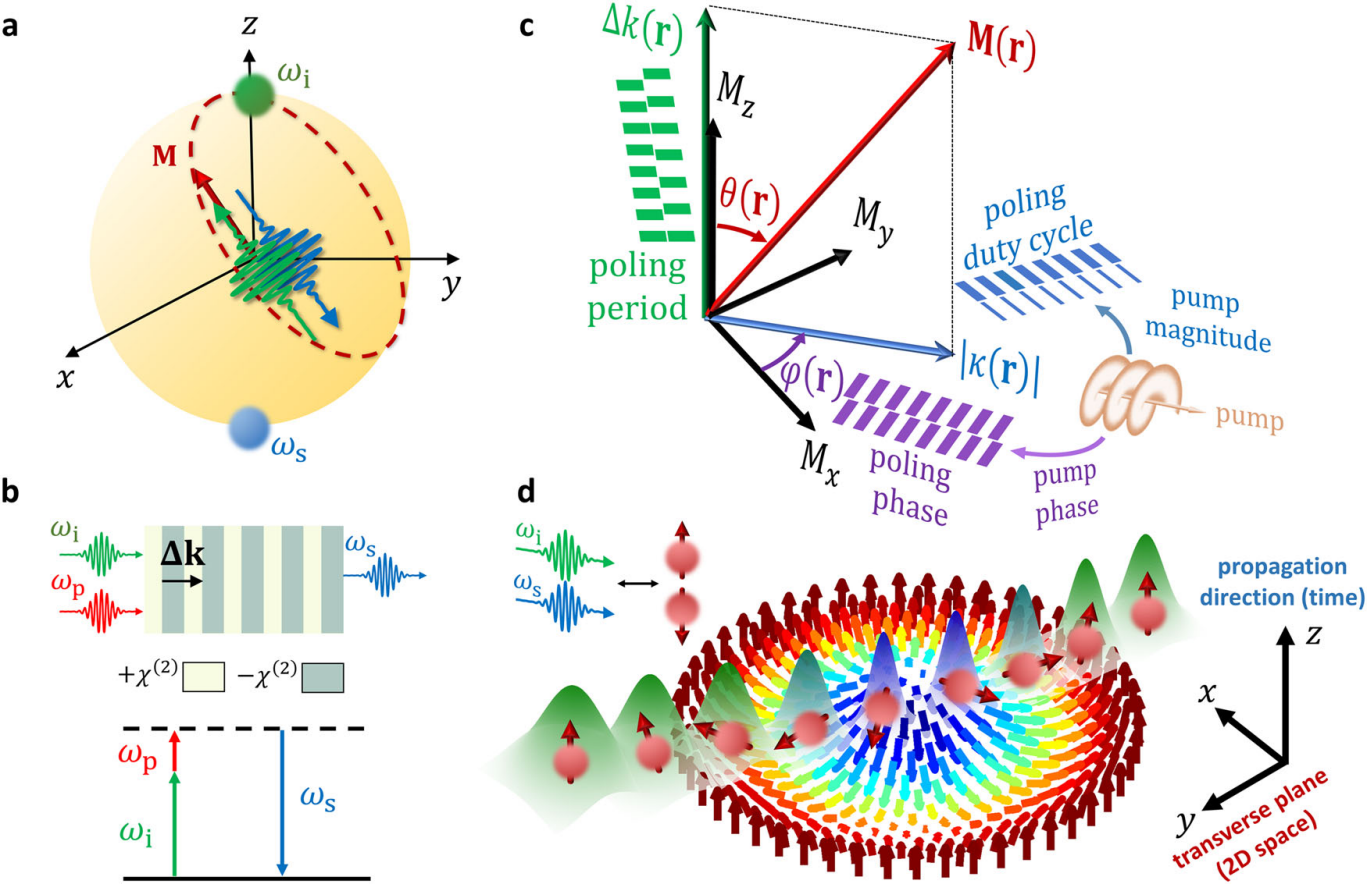
Emulation of spin transport phenomena in nonlinear optics. **a** Bloch sphere representation of a two-level system—here comprising the idler ($\omega _{\mathrm{i}}$) and signal ($\omega _{\mathrm{s}}$) frequencies—wherein the frequency degree of freedom acts as a pseudospin dimension. Consequently, an effective magnetization field ${\mathbf{M}}$, driving the dynamics, can be defined. **b** Realization of two-frequency dynamics. In sum-frequency generation, the idler and pump photons generate a higher-frequency signal photon. For an undepleted pump, the interaction reduces to a two-level system of $\omega _{\mathrm{i}}$ and $\omega _{\mathrm{s}}$. A periodic spatial modulation of the nonlinearity compensates for the momentum mismatch ${\mathrm{{\Delta} }}k$. **c** Parameter space defining the spatially-varying effective magnetization, ${\mathbf{M}}\left( {\mathbf{r}} \right)$. The *z*-component is controlled by the momentum mismatch ${\mathrm{{\Delta} }}k\left( {\mathbf{r}} \right)$—namely, the poling period (marked in green). The radial component is defined by the nonlinear coupling strength $\left| {\kappa ({\mathbf{r}})} \right|$, proportional to the modulation duty cycle and to the pump field strength (marked in blue). The polar angle $\varphi \left( {\mathbf{r}} \right)$ is given by the relative phase between the poling phase and pump phase front (marked in purple). $\theta \left( {\mathbf{r}} \right)$ denotes the elevation angle. All these parameters are tuneable and can be used to tailor arbitrary magnetization textures ${\mathbf{M}}\left( {\mathbf{r}} \right)$. **d** Dynamics of the pseudospin and position of the light beam as it traverses a synthetic magnetization texture. In the photonic system, the propagation coordinate represents time, and the location of the light beam on the transverse plane is analogous to the position of the spin-1/2 particle (axis inset), while the spin degree of freedom is analogous to the color of the light (green/blue colors representing spin up/down). In a similar manner to the electron spin, adiabatically following the local magnetization direction, light undergoes adiabatic frequency conversion as it propagates.

**Fig. 2:**
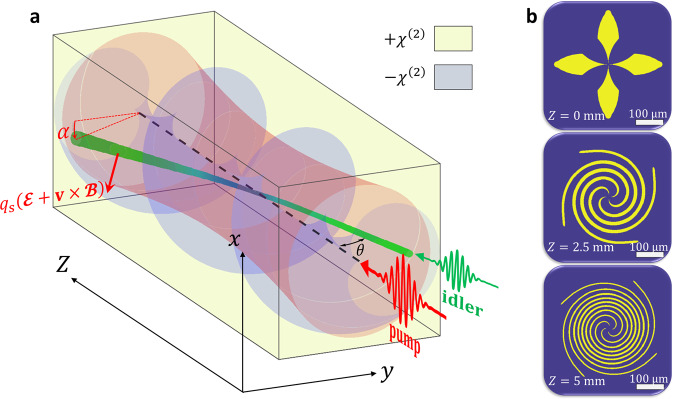
Topological Hall effect for light beams. **a** A quadratic $\left( {\chi ^{\left( 2 \right)}} \right)$ nonlinear photonic crystal with an imprinted synthetic skyrmion texture (gray and yellow for negative and positive nonlinearity poling) is illuminated by a broad pump beam (red). A tightly focused beam in the idler frequency (green) impinges the crystal at an angle $\theta$ with respect to the optical axis (dashed black line). As the beam propagates, it experiences adiabatic frequency conversion to the signal frequency (blue) and back to the idler frequency, as the spectral pseudospin adiabatically follows the local direction of the effective skyrmionic magnetization (see also Fig. 1d). As a result, a real-space geometric phase is accumulated, and the synthetic gauge fields associated with it give rise to an emergent Lorentz force, causing the beam waist to deflect by an angle $\alpha$ in the transverse plane (shifting the *x*-axis position of the beam at the output). **b** cross sections of a high-order skyrmionic nonlinear photonic crystal used in Fig. 3c,d, at crystal center $(Z = 0)$, $Z = 2.5\,{\mathrm{mm}}$, and $Z = 5\,{\mathrm{mm}}$. The nominal quasi-phase-matching period is $10.24\,{\mathrm{\mu}} {\mathrm{m}}$, and it changes between $10.33\,{\mathrm{\mu}} {\mathrm{m}}$ at the crystal center (the optical axis), to $10.15\,{\mathrm{\mu}} {\mathrm{m}}$ at the edges.

Emulating 2D spin transport necessitates a pseudospin degree of freedom, an effective magnetization field acting on the pseudospin, and a space-time along which the dynamics is probed. We define the pseudospin degree of freedom by considering a nonlinear optical process involving two interacting frequencies, which can be geometrically represented on a Bloch sphere^25,32,44^ (Fig. 1a–b). This degree of freedom is controlled by the inherent phase matching of the process and its complex coupling coefficient, which together define an effective magnetization field applied on the pseudospin^33,45^ (Fig. 1c). Considering the propagation direction as a time axis, the transverse profile of a light beam defines the wavefunction of a massive particle in two spatial dimensions (see Figs. 1d and 2a), with its dynamics dictated by the variation of the effective magnetization field in space.

Full control over the dynamics is achieved by engineering the nonlinearity in the material, causing the detuning from phase matching and the complex coupling strength to change in space, for a fixed pump illumination. Alternately, the complex coupling can be tailored by shaping the pump field, together with a correspondingly engineered variation in the detuning. The nonlinear process and its tunable parameters are presented in Fig. 1b-c.

In what follows, we consider the three-wave mixing process of sum-frequency generation in a quadratic nonlinear photonic crystal between an idler (*E*_*i*_), signal (*E*_*s*_) and pump (*E*_*p*_) electric fields (Fig. 1b). Assuming the pump field is strong and nondepleted, the interplay is effectively only between the idler and signal fields. In this setting, the transverse propagation of the light beam and its frequency (idler or signal) emulate the motion of a spin-1/2 particle at a certain spin state, traversing a two-dimensional magnetization texture (see Fig. 1d).

Under the long pump wavelength approximation^46^, the paraxial coupled wave equations for the signal and idler slowly varying envelopes are:

1 $$i\frac{\partial }{{\partial Z}}\left( {\begin{array}{*{20}{c}} {E_{\mathrm{i}}} \\ {E_{\mathrm{s}}} \end{array}} \right) = \left[ {\frac{{{\mathbf{p}}_{\mathrm{T}}^2}}{{2\bar k}} - \left( {\begin{array}{*{20}{c}} 0 & {\kappa ^ \ast e^{ - i{\mathrm{{\Phi} }}}} \\ {\kappa e^{i{\mathrm{{\Phi} }}}} & 0 \end{array}} \right)} \right]\left( {\begin{array}{*{20}{c}} {E_{\mathrm{i}}} \\ {E_{\mathrm{s}}} \end{array}} \right)$$

where $\kappa = 2d_{{\mathrm{eff}}}\bar \omega ^2E_{\mathrm{p}}/\bar kc^2$ is the nonlinear coupling constant; *d*_eff_ is the corresponding component of the effective nonlinearity tensor; $\bar k,\bar \omega$ are the mean wavenumber and frequency for the idler and signal fields; *c* is the speed of light in vacuum; ${\mathbf{p}}_{\mathrm{T}} = - i{\mathbf{\nabla}} _{\mathrm{T}}$ and ${\mathbf{r}}_{\mathrm{T}} = \left( {x,y} \right)$ are the transverse momentum operator and position vector, respectively; *Z* is the propagation coordinate; and ${\mathrm{{\Phi} }}\left( {\mathbf{r}} \right) = {\int}_0^Z {{\Delta} k\left( {{\mathbf{r}}_{\mathrm{T}},Z^{\prime} } \right)dZ^{\prime} }$ is the phase mismatch accumulated along the propagation (${\mathrm{{\Delta} }}k\left( {\mathbf{r}} \right)$ is the position-dependent momentum mismatch, defined in Fig. 1b).

A local gauge transformation to the rotating frame can be applied to Eq. (1) by defining $U\left( {\mathbf{r}} \right) = {\mathrm{diag}}\left( {e^{ - i{\mathrm{{\Phi} }}\left( {\mathbf{r}} \right)/2},e^{i{\mathrm{{\Phi} }}\left( {\mathbf{r}} \right)/2}} \right)$, while rewriting it for the transformed two-component field vector ${\mathrm{{\Psi} }} = U^\dagger \left( {E_{\mathrm{i}},E_{\mathrm{s}}} \right)^T$:

2 $$i\frac{\partial }{{\partial Z}}{\mathrm{{\Psi} }} = \left[ {\frac{{\left( {{\mathbf{p}}_{\mathrm{T}} - {\mathbf{{\cal{A}}}}} \right)^2}}{{2\bar k}} - {\mathbf{\sigma }} \cdot {\mathbf{M}}} \right]{\mathrm{{\Psi} }},$$

where ${\mathbf{M}} = ({\mathrm{Re}}\,\kappa ,{\mathrm{Im}}\,\kappa ,{\mathrm{{\Delta} }}k/2)$ is the effective magnetization operator, ${\boldsymbol{\sigma }} = \left( {{{\sigma }}_x,{{\sigma }}_y,{{\sigma }}_z} \right)$ is the Pauli matrix vector and ${\mathbf{{\cal{A}}}} = iU^\dagger {\mathbf{\nabla}} _{\mathrm{T}}U = \frac{1}{2}{\mathbf{\nabla}} _{\mathrm{T}}{\mathrm{{\Phi} }}{\mathbf{\sigma }}_z$ is the vector potential operator. The similarity between Eq. (2) and the Pauli-Schrodinger equation—describing a spin-1/2 particle in a magnetic field—is what allows us to describe the optical fields in terms of spin currents propagating in a magnetization texture. Most importantly, the effective magnetization vector ${\mathbf{M}}$ in the pseudospin space can be varied according to the crystal design and pump field shape (see Fig. 1c), thus enabling control over the spin transport. With recent advances in fabricating three-dimensional NLPCs^35–38^, the necessary degrees of freedom for fully controllable engineering of the parameter space are now available. Therefore, the formalism given by Eq. (2) constitutes a general framework to explore the dynamics of 2D spin-1/2 particles influenced by arbitrary magnetization textures using nonlinear optics.

### The topological Hall effect for light beams

We demonstrate the capabilities of our approach by emulating the topological Hall effect (THE), in which polarized spin currents are deflected by a topologically nontrivial magnetization texture (such as magnetic skyrmions^11–13^). In the adiabatic regime of the THE, the orientation of electron spin follows the local normalized magnetation direction ${\hat{\mathbf{M}}} = {\mathbf{M}}/\left| {\mathbf{M}} \right|$, causing the electron wavefunction to deflect due to an acquired geometric phase (illustrated in Fig. 1d).

We implement the local gauge transformation $U^{\prime} \left( {\mathbf{r}} \right) = \exp \left[ { - i\theta \left( {\mathbf{r}} \right){\boldsymbol{\sigma }} \cdot {\hat{\boldsymbol{\varphi }}}\left( {\mathbf{r}} \right)/2} \right]$ on the spinor wavefunction ${\mathrm{{\Psi} }}$ of Eq. (2), aligning the synthetic optical spin with the local magnetization direction^11,47^. Here, $\theta \left( {\mathbf{r}} \right) = \arccos {\hat{\mathbf{M}}} \cdot {\hat{\mathbf{z}}}$ and ${\hat{\boldsymbol{\varphi }}}\left( {\mathbf{r}} \right) = {\hat{\mathbf{z}}} \times {\hat{\mathbf{M}}}/\left| {{\hat{\mathbf{z}}} \times {\hat{\mathbf{M}}}} \right|$, are the elevation angle and polar unit vector, as defined in Fig. 1c. The transformed state ${\mathrm{{\Psi} }}^{\prime} = U^{\prime \dagger }{\mathrm{{\Psi} }}$ then satisfies the equation of motion:

3 $$i\frac{\partial }{{\partial Z}}{\mathrm{{\Psi} }}^{\prime} = \left[ {\frac{{\left( {{\mathbf{p}}_{\mathrm{T}} - {\mathbf{{\cal{A}}}}^{\prime} } \right)^2}}{{2\bar k}} + {\cal{V}}} \right]{\mathrm{{\Psi} }}^{\prime} ,$$

where the new vector and scalar gauge potentials are ${\mathbf{{\cal{A}}}}^{\prime} = iU^{\prime \dagger }{\mathbf{\nabla}} _{\mathrm{T}}U^\prime + U^{\prime \dagger }{\mathbf{{\cal{A}}}}U^{\prime}$ and ${\cal{V}} = - iU^{\prime \dagger }\partial _ZU^\prime - M\sigma _z$. To describe the spatial dynamics, we derive the synthetic electric and magnetic fields from the gauge potentials. In the adiabatic regime, the dynamics for the two spinor eigenstates is decoupled, and for each frequency eigenvalue we associate an effective charge $q_{\mathrm{s}} = \pm 1$, depending on the orientation of its associated pseudospin with respect to the local effective magnetization (parallel or anti-parallel). Assuming the magnetization is constant in the propagation direction $\left( {\partial _Z{\mathbf{M}} = 0} \right.$, which implies time invariance in the electronic system, see the Supplementary Material):

4a $${\mathbf{{\cal{E}}}} = - {\mathbf{\nabla}} _{\mathrm{T}}{\cal{V}} - \partial _Z{\mathbf{{\cal{A}}}}^{\prime} = \frac{{M_{\mathrm{T}}{\mathbf{\nabla}} _{\mathrm{T}}M_{\mathrm{T}}}}{M},$$

4b $${\mathbf{{\cal{B}}}} = {\mathbf{\nabla}} _{\mathrm{T}} \times {\mathbf{{\cal{A}}}}^{\prime} = - \frac{1}{2}{\hat{\mathbf{z}}}\left[ {{\hat{\mathbf{M}}} \cdot \left( {\partial _x{\hat{\mathbf{M}}} \times \partial _y{\hat{\mathbf{M}}}} \right)} \right],$$

where $M_{z}^{2} + M_{\mathrm{T}}^{2} = M^{2}$, with $M_{z}, M_{\mathrm{T}}$ denoting the longitudinal (out-of-plane) and transverse (in-plane) components of the magnetization, respectively. As such, each eigenstate experiences an *opposite* Lorentz-like force, given by:

5 $${{{\cal{F}}}} = q_{\mathrm{s}} ({{\cal{E}}} + {\mathbf{v}} \times {{\cal{B}}}),$$

where ${\mathbf{v}} = {\mathbf{k}}_{\mathrm{T}}/\bar k,$ the beam angle with respect to the optical axis, serves as an effective velocity of the light beam in the transverse plane. This concept is illustrated in Fig. 2, depicting a proposed experimental system: a broad pump beam covering the entire facet of a NLPC, while an idler (or signal) beam is tightly focused inside the crystal—acting as a localized spin wavepacket in the transverse plane (see also Fig. 1d). The relative ease in which one can control the incidence angle and beam size of light, effectively changing the spin wavepacket’s shape and velocity, is a main strength of our approach to spin transport emulation.

The synthetic magnetic field in Eqs. (4) and (5) is directly related to the geometric phase acquired during propagation of light through the crystal, and it’s magnitude is solely dependent on the real-space Berry curvature of the magnetization^47^. As such, only topologically nontrivial magnetization textures can induce a deflection of the beam trajectory associated with the THE (see Supplementary Material), with the so-called skyrmion number $S = {\int} {d^2{\mathbf{r}}\widehat {\mathbf{M}} \cdot ( {\partial _x{\hat{\mathbf{M}}} \times \partial _y\widehat {\mathbf{M}}} )/4\pi\, \ne\, 0}$ as the relevant topological invariant. On the other hand, the synthetic electric field term in Eqs. (4) and (5) depends on the coupling strength explicitly through $M_{\mathrm{T}} \propto \kappa$, and is thus related to the dynamic phase accumulated during the interaction (leading, in the fully phase-matched case, to a photonic Stern-Gerlach effect^45,46^ wherein ${\cal{F}} = q_{\mathrm{s}}{\mathbf{\nabla}} _{\mathrm{T}}M_{\mathrm{T}}$).

### Simulating the topological Hall effect from single skyrmions with light

Using our formalism, it is possible to explore the topological Hall effect occurring in otherwise thermodynamically unstable magnetization textures, such as single high-order skyrmions^14^. To this end, let us define a circularly-symmetric magnetization texture with a single topological defect as follows:

6 $$\widehat {\mathbf{M}}\left( {\rho ,\phi } \right) = \sqrt {1 - m^2\left( \rho \right)} \left[ {\cos \left( {n\phi + \eta } \right)\widehat {\mathbf{x}} + \sin \left( {n\phi + \eta } \right)\widehat {\mathbf{y}}} \right] + m\left( \rho \right)\widehat {\mathbf{z}},$$

where $\rho ,\phi$ are the (radial and azimuthal) transverse polar coordinates; $- 1 \le m\left( \rho \right) = {\Delta} k\left( \rho \right)/2M_0 \le 1$ is a (normalized) radial variation of the phase mismatch, accompanied by a radial variation $\sqrt {1 - m^2\left( \rho \right)}$ in the nonlinear coupling strength $\left| \kappa \right|;\,M_0$ is a constant magnitude of the magnetization vector such that ${\mathbf{M}} = M_0\widehat {\mathbf{M}}$; $\eta$ is a constant phase factor; and $n$ is a chosen winding number, which we consider for now to be induced solely by the chirality of the NLPC (see Supplementary Material for further details).

For the given magnetization in Eq. (6), the synthetic electric and magnetic fields are:

7a $${\mathbf{{\cal{E}}}} = - M_0m\left( \rho \right)\frac{d}{{d\rho }}m\left( \rho \right)\widehat {\mathbf{\rho }},$$

7b $${\mathbf{{\cal{B}}}} = - \frac{1}{2}\frac{n}{\rho }\frac{d}{{d\rho }}m\left( \rho \right)\widehat {\mathbf{z}},$$

with the skyrmion number $S = \left[ {m\left( R \right) - m\left( 0 \right)} \right] \times (n/2)$, where *R* is the radius of the entire magnetization domain. Note that only the magnetic field is directly dependant on the winding number *n*, nullifying its contribution for any topologically trivial magnetization. As a consequence of Eq. (5), the electric field contribution to the synthetic Lorentz force acts now as an attractive (or repulsive) central force for opposite pseudospins, whereas the magnetic deflection of the beam also changes its sign depending on the corresponding eigenstate.

Though quite a specific solution, Eqs. 6,7 are still general enough such that the simulation of several meaningful magnetization textures may be achieved, through the simple task of guessing the distribution function $m\left( \rho \right)$, winding number $n$ and the constant phase $\eta$. For example, a Néel-type skyrmion^48^ (or Néel-type antiskyrmion) can be described by $m\left( \rho \right) = \mp \cos (\pi \rho /R)$, winding number $n = \pm 1$, and phase factor $\eta = 0$. Similarly, changes can be made to accommodate Bloch-type skyrmions^48^ ($\eta = \pi /2$), or high-order skyrmions ($\left| n \right| > 1$).

**Fig. 3:**
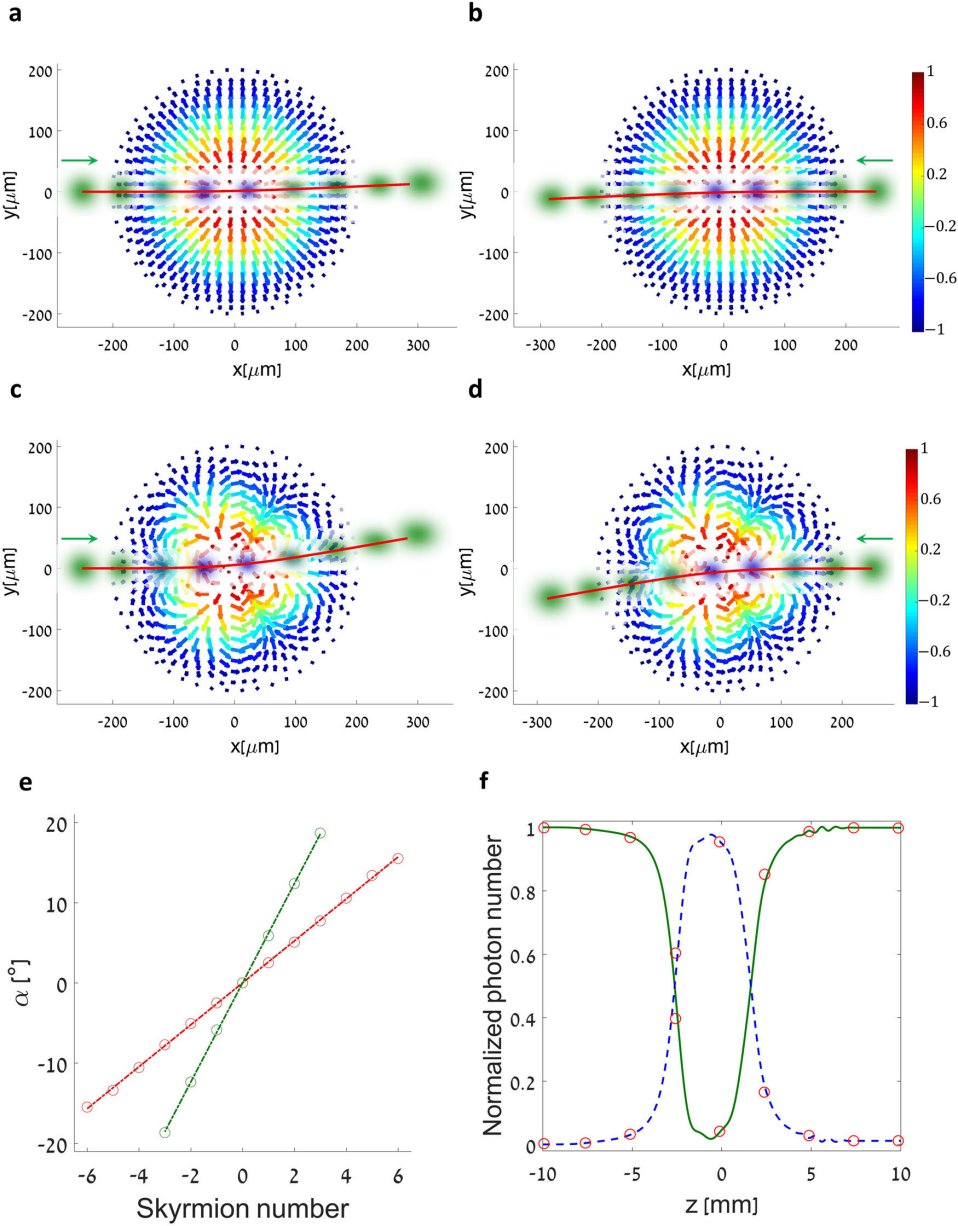
Simulating the topological Hall effect in skyrmionic nonlinear photonic crystals. **a, b** Simulated beam shape and position in the transverse plane of the crystal, for selected depths of propagation inside the NLPC (the successive points are for $z$ locations of $- 10.0, - 7.5, - 5.0, - 2.5,0,2.5,5.0,7.5$ and $10.0\,{\mathrm{mm}}$). The theoretical trajectory of the beam’s center-of-mass motion is depicted by solid red lines. In **a**, an idler beam enters the NLPC at an entrance angle of 1.5 degrees, traversing a Néel-type antiskyrmion ($S = - 1$, $m\left( \rho \right) = \cos (\pi \rho /R),n = 1,\eta = 0,R = 200\,{\mathrm{\mu}} {\mathrm{m}}$) from left to right. Also inset is the transverse component of the synthetic magnetization in the NLPC, color-coded by its corresponding $z$-component. In **b**, the dynamics are flipped (from right to left), and as a result, the beam is deflected in the opposite direction—a clear signature of the THE. **c**, **d** Same as in **a**, **b**, but with an engineered high-order antiskyrmion $(n = 4)$, causing a more pronounced difference in the deflection. **e** Deflection angle of the beam for varying skyrmion numbers. By considering the rotation of the velocity vector ${\boldsymbol{v}}$ by the magnetic field, the deflection angle of the beam’s center-of-mass should be $\alpha \propto S/R\left| {\boldsymbol{v}} \right|$ (see Supplementary Material). Simulation results show a clear linear dependence on the skyrmion number, for two different cases (red circles—$R = 200\,{\mathrm{\mu}} {\mathrm{m}}$, $\left| {\boldsymbol{v}} \right| = \sin \left( {1.5^\circ } \right)$; green circles - $R = 100\,{\mathrm{\mu}} {\mathrm{m}}$ and $\left| {\boldsymbol{v}} \right| = \sin \left( {1.2^\circ } \right)$). **f** Photon number, normalized by its initial value, in the idler (green continuous line) and signal (blue dashed line) frequencies as a function of the propagation inside the NLPC for case **a**, demonstrating adiabatic frequency conversion. Full simulation parameters are given in the Methods.

Numerical simulations of nonlinear beam propagation inside a skyrmionic NLPC, based on the split-step Fourier method (see Methods), are presented in Fig. 3. We note that in the numerical calculation we only apply standard assumptions, such as reflection-less, paraxial propagation, rather than the approximations employed for the analytical solution. Figure 3a,b shows the idler beam’s transverse dynamics as it propagates through a crystal with a skyrmion magnetization of $S = - 1$: for opposite incident angles, the beam deflects to opposite transverse directions, in a clear manifestation of the THE^11,47^. A reversed angle is analogous to a reversed velocity, and the opposite deflection is intimately connected to the nonreciprocity induced by the synthetic magnetic field.

The effect becomes more pronounced for higher skyrmion numbers or smaller skyrmion radii, as illustrated in Fig. 3c–e, and in Supplementary Movies 1 and 2. Our theoretical model seems to describe the nonlinear optical system well, as evident by the predicted trajectories (red lines in Fig. 3a–d) and by the adiabatic frequency conversion during propagation (Fig. 3f), in complete analogy to the electron spin in the THE. The signal beam behaves similarly, although it is deflected to the opposite direction, as expected (see the Supplementary Material).

### Domain walls engineering

**Fig. 4:**
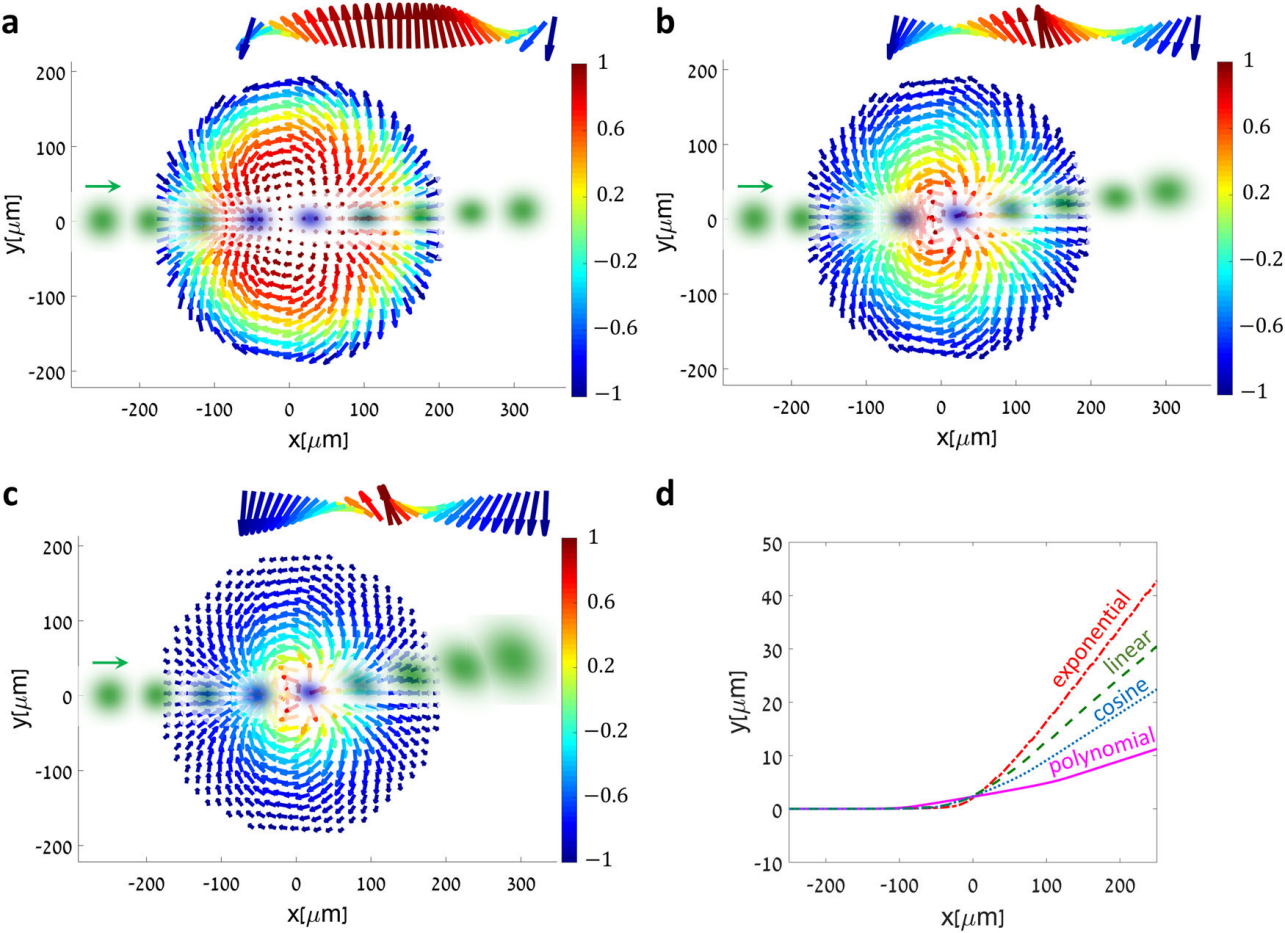
Tailoring domain walls to engineer the topological Hall effect for light. Simulated beam shape and position in the transverse plane of the crystal, for selected depths of propagation inside the NLPC and different domain wall distributions ($R = 200\,{\mathrm{\mu}} {\mathrm{m}},n = 2,\eta = 0$, and beam angle $1.5^\circ$ in all panels; insets show the domain wall cross sections). **a** cubic polynomial dependence $m\left( \rho \right) = 1 - 2\left( {\rho /R} \right)^3$ (with ${\cal{B}} \propto \rho$). **b** linear dependence $m\left( \rho \right) = 1 - 2\rho /R$ (with ${\cal{B}} \propto 1/\rho$). **c** exponential dependence $m\left( \rho \right) = {\mathrm{exp}}( - \rho /R)[1 - (\rho /R)(1 + e)]$ (with ${\cal{B}} \propto {\mathrm{exp}}( - \rho /R)/\rho$). Inset is the transverse component of the synthetic magnetization in the NLPC, color-coded by its corresponding $z$-component. It appears that the deflection becomes more dominant as the domain wall transition effectively occurs in a smaller area. **d** Simulated center-of-mass trajectories of the light beams traversing the different domain walls of **a**–**c**, as compared to a conventional Néel-type skyrmion ($m\left( \rho \right) = \cos (\pi \rho /R)$, with ${\cal{B}} \propto {\mathrm{sinc}}\left( {\pi \rho /R} \right)$).

Interestingly, the dynamics in the THE regime can be significantly altered by the exact variation of the magnetization from one out-of-plane direction to its opposite—called the domain wall. The ability to accurately engineer 3D features of NLPCs allows us to precisely design domain walls, including the adjustment of the skyrmion winding, size and radial profile $m\left( \rho \right)$. Figure 4a–c shows the simulated transverse dynamics of light beams propagating through $S = - 2$ crystals with cubic (Fig. 4a), linear (Fig. 4b), and exponential (Fig. 4c) domain walls (see also Supplementary movies 3 and 4).

While all three configurations possess the same radius and topological invariant, they exert a different force on the light beams, resulting in different center-of-mass trajectories. The trajectories are compared in Fig. 4d, along with that found in conventional Néel-type skyrmions with cosine domain walls. Evidently, topologically equivalent magnetic textures yield different THE signatures, with the underlying mechanism being the strong singularity emerging in the synthetic magnetic field distribution (Fig. 4b, c). Interestingly, a larger and more localized Berry curvature enhances the Lorentz force, similarly to how a larger local optical angular-momentum density increases the torque applied on particles by optical vortices^49^, even though both effects initially stem from global topological charges.

### Active all-optical control over the topological Hall effect with light

**Fig. 5:**
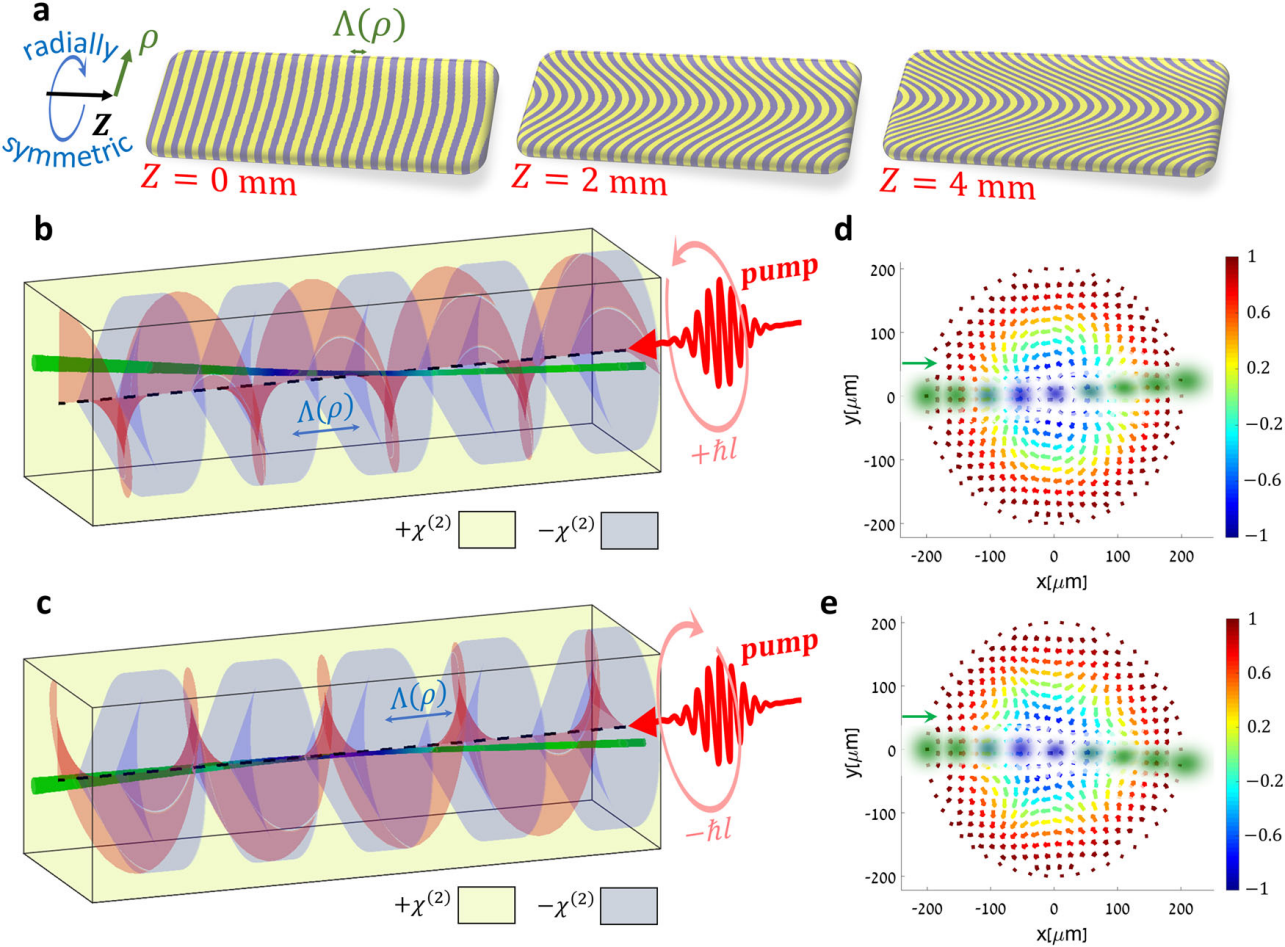
All-optical angular-momentum control of the topological Hall effect for light. **a** Cross section of the three-dimensional nonlinear photonic crystal along the optical axis, showing the radial variation in the modulation period, ${\Lambda} \left( \rho \right)$ ($\rho$ denotes the radial coordinate with respect to the optical axis). The nominal quasi-phase-matching period ${\Lambda} \left( \rho \right)$ is $10.24\,{\mathrm{\mu}} {\mathrm{m}}$, and it changes between $10.33\,{\mathrm{\mu}} {\mathrm{m}}$ at the crystal center, to $10.15\,{\mathrm{\mu}} {\mathrm{m}}$ at the edges. Note that in this case the required $\chi ^{\left( 2 \right)}$ pattern is rotationally symmetric, given by $\chi ^{\left( 2 \right)} = {\mathrm{sign}}\left\{ {\cos \left[ {2\pi Z/{\Lambda} \left( \rho \right)} \right]} \right\}$. **b**, **c** a quadratic ($\chi ^{\left( 2 \right)}$) nonlinear photonic crystal with a radially symmetric variation in the periodicity is illuminated by a broad pump beam carrying orbital angular momentum of $\pm \hbar l$ (red). The pump beam profile and phase front, together with the 3D NLPC structure, induce a topologically nontrivial magnetization texture, with opposite skyrmion numbers $S = \pm l$. As a result, an incident idler beam is deflected according to the topological Hall effect. **d**, **e** Simulation results for the optically-controlled topological Hall effect, for a pump beam carrying OAM of **d** $+ 2\hbar$ and **e** $- 2\hbar$, with a waist of $w_0 = 100\,{\mathrm{\mu}} {\mathrm{m}}$ ($S = \pm 2$, $n = l = \pm 2,\eta = 0,R = 200\,{\mathrm{\mu}} {\mathrm{m}}$ and beam angle of $1.1^\circ$). The idler beam is deflected in opposite directions according to the skyrmion number.

Effective magnetization textures in nonlinear optical media can also be induced directly by light, enabling diverse opportunities for all-optically-controlled devices. In the context of generating effective skyrmion magnetizations, pump fields carrying orbital angular momentum^49^ (OAM) can provide the required chirality^25^ and coupling strength variation, through their phase and intensity profiles (Fig. 1c). Thus, the NLPC design is greatly simplified, requiring only a radially varying periodicity (see Fig. 5a).

In this manner, the pump and crystal together induce an effective skyrmion of order $S = l$, where $\hbar l$ is the pump OAM, whereas active control over the THE is enabled by changing the OAM. Examples are given in Fig. 5, where we simulate the THE from high-order skyrmions with Gauss-Laguerre pump beams carrying OAM of $\pm 2\hbar$. As expected, it appears that for opposite OAM values, the deflection is reversed, in accordance with the skyrmion number changing its sign.

Aside from spatially modulating the pump, temporal modulation can also bring about new degrees of control. Since optical nonlinear effects relax in ultrafast time scales, modulation frequency should only be limited by the propagation time through the NLPC, allowing working rates on the order of tens of GHz for the crystal lengths considered in this work. The simplest modulation is, of course, mere intensity modulation, which turns the THE on or off. However, it is also possible to modulate the angular momentum of the pump in time, thus changing between the deflection properties of the THE.

## Discussion

In summary, we presented a framework to connect the fields of spintronics and nonlinear optics through the use of 3D nonlinear photonic crystals, showing how any spin transport through any 2D magnetization texture may now be emulated by light. As an example, we realized the topological Hall effect for light via effective skyrmion textures with different topologies and domain wall distribution, while showing the capability for all-optical control. Our framework can readily simulate periodic^7,8,50,51^ or disordered^2^ magnetization textures, but more importantly—emulate hard-to-implement quantum spin phenomena via quantum signal/idler light. Such phenomena include Anderson localization^41^ of spinors or their quantum random walks^42^, utilizing single-photon sources; or entangled spin transport, as in superconducting spintronics^43^, using multiphoton frequency-entangled states^52^.

Further extending our formalism may allow the simulation of scenarios where a direct experiment or numerical calculation are impractical. Such is the case when introducing Kerr nonlinearity, which could promote effective many-body interactions between pseudospins^53^; or cascaded nonlinear interactions, enabling the simulation of higher spin Hilbert spaces^46^. Even simple extensions, such as time-variance ($\partial _Z{\mathbf{M}}\, \ne\, 0$), can increase the range of effects produced by our system to enable the study of transient phenomena such as melting^39^ or spin waves^40^. Allowing pump depletion^54^, i.e., permitting the pump intensity to be affected by the signal/idler waves, will describe the full nonlinear dynamics under which Berry curvature is known to persist^55^, but will complicate the calculations. In the latter scenario, the effective magnetization will be perturbed by the pseudospin current, much like in spin-transfer torque^56^.

Novel ideas for both classical and quantum optical information processing can now benefit from decades of spintronics research, as methods and devices to control spin current may be used to direct optical flow. For example, a practical application for the all-optical modulation of a skyrmionic NLPC can be a relatively broadband^32^ optical router, operating in tens of GHz, for either classical optical communications or to control quantum frequency combs^52^—an emerging candidate for quantum information processing. Skyrmionic NLPCs could also serve as multi-level logic gates (or single-qubit gates for frequency-entangled states), where the pump OAM, or even its polarization, contain the information. Overall, the system is expected to display topological robustness for deviations in pump power, input wavelengths and poling, as was demonstrated in earlier observations of adiabatic processes in nonlinear optics^32–34,57^.

The experimental realization of our proposed system is fast-approaching, considering the rate of advancement in fabricating 3D NLPCs. Rather than feature resolution, which is quite sufficient^35^, the main challenge imposed by our system is the long crystal length $L$ necessary to ensure the adiabatic condition^33^ $L \gg 2\pi /\kappa$. The nonlinear coupling $\kappa$ can be increased with pump peak intensity, allowing for shorter crystal lengths, though it is still limited by the material damage threshold. Another route to decreasing the crystal length is by relaxing the adiabaticity of the system, which maintains geometric phase effects even for $L\sim 2\pi /\kappa$^34^. Finally, much shorter crystal lengths can be achieved by using 3D nonlinear cavities and/or waveguides^36^, thanks to the large field confinement, though further research is required to discover analogous spintronic and topological phenomena in such systems.

## Methods

Simulations were performed using a split-step Fourier^58^ method, where the propagation of fields is calculated under the paraxial approximation. Idler and signal fields were initialized as Gaussian beams at $z = - 10\,{\mathrm{mm}}$, and were then allowed to propagate through the crystal. The pump was assumed to retain its given spatial mode (either Gaussian or Gaussian-Laguerre mode; pump beam waists corresponding to the different simulations are explicitly mentioned in the text). The simulated wavelengths were chosen as $\lambda _{\mathrm{i}} = 532\,{\mathrm{nm}}$, $\lambda _{\mathrm{s}} = 480\,{\mathrm{nm}}$ for the idler and signal, respectively, and $\lambda _{\mathrm{p}} = 5\,\mu {\mathrm{m}}$ for the pump field, with a pump peak intensity of $153\,{\mathrm{MWcm}}^{ - 2}$, which is quite possible with commercially available laser systems. The nonlinear medium was a bulk ${\mathrm{LiNbO}}_3$ $\left( {d_{33}\sim 23.4\,{\mathrm{pmV}}^{ - 1}} \right)$ crystal, $20\,{\mathrm{mm}}$ in length. Both idler and signal beams were initially focused onto the crystal center, with a minimal waist of $25\,\mu {\mathrm{m}}$ and at an angle $\theta = k_x/k$ (specified in the main text) with respect to the optical axis. All parameters were optimized to ensure adiabaticity of the nonlinear interaction^57^, such that the adiabatic change is slower than the system’s Rabi oscillations, namely $\kappa L \gg 2\pi$. For the parameters simulated, $\kappa L = 56$ (see Fig. 3f). All parameters were chosen within experimentally available regimes for real materials and laser sources. Our use of a mid-IR pump in the simulation is a favorable approximation^45^, which ensures that the wavevectors of the idler and signal are similar, but this is not crucial for an experiment, as even with far less intensity and a far shorter pump wavelength ($\lambda _{\mathrm{p}} = 1064\,{\mathrm{nm}}$), spatially dependent nonlinear geometric phase effects were observed^34^.

## Supplementary information

Supplementary Information

Description of Additional Supplementary Files

Supplementary Movie 1

Supplementary Movie 2

Supplementary Movie 3

Supplementary Movie 4

## Data Availability

The data supporting the findings of this study are available from the corresponding author upon reasonable request.

## Code availability

The code supporting the plots within this paper are available from the corresponding author upon reasonable request.
