# Supplementary material for: Emulating spin transport with nonlinear optics, from high-order skyrmions to the topological Hall effect
Source: Nat Commun. 2021 Feb 17;12:1092. doi: 10.1038/s41467-021-21250-z (PMC7889664; doi:10.1038/s41467-021-21250-z)
Supplement: Supplementary file 1 — Supplementary Information [file 41467_2021_21250_MOESM1_ESM.pdf]

<u>Supplementary Information - Emulating spin transport with nonlinear optics, from high-order skyrmions to the topological Hall effect</u>

Karnieli et. al.

Table of contents

Supplementary Note 1: Derivation of the topological Hall effect dynamics

Supplementary Note 2: Derivation of the synthetic gauge fields

Supplementary Note 3: Single skyrmion magnetization texture

Supplementary Note 4: Simulation results for the signal field

Supplementary Note 5: Topological deflection: in-plane vortex vs skyrmion

Supplementary Note 6: Classical particle dynamics model

Supplementary Note 7: Crystal structure

Supplementary Note 8: Simulation videos: Left-to-right and right-to-left propagation in a Neel-type skyrmion of order $S = 4$; Propagation in a tailored domain wall skyrmion of $S = 2$ (linear and cubic variations).

Supplementary Note 1: Derivation of the topological Hall effect dynamics

The coupled wave equations in a sum frequency generation process with undepleted pump are[1]:

$$i\frac{\partial E_{\mathrm{i}}}{\partial Z} = -\frac{\nabla_{\mathrm{T}}^2}{2k_{\mathrm{i}}}E_{\mathrm{i}} - \frac{2d_{\mathrm{eff}}\omega_{\mathrm{i}}^2}{k_{\mathrm{i}}c^2}E_{\mathrm{p}}^* E_{\mathrm{s}} e^{-i\Phi(\mathbf{r})}, \qquad (1)$$

$$i\frac{\partial E_{\mathrm{s}}}{\partial Z} = -\frac{\nabla_{\mathrm{T}}^2}{2k_{\mathrm{s}}}E_{\mathrm{s}} - \frac{2d_{\mathrm{eff}}\omega_{\mathrm{s}}^2}{k_{\mathrm{s}}c^2}E_{\mathrm{p}} E_{\mathrm{i}} e^{i\Phi(\mathbf{r})}, \qquad (2)$$

where

$$\Phi(\mathbf{r}) = \int_0^Z \Delta k(\mathbf{r}_T, Z')dZ'\,, \qquad (3)$$

is the accumulated phase mismatch.

Working in the long wavelength pump approximation ($\lambda_{\mathrm{p}} \gg \lambda_{\mathrm{i}}, \lambda_{\mathrm{s}}$), we can assume $k_{\mathrm{i}} \sim k_{\mathrm{s}} \sim \bar{k}$ and define the coupling strength $\kappa = \frac{2d_{\mathrm{eff}}\omega^2}{\bar{k}c^2}E_{\mathrm{p}}$, we then rewrite the coupled wave equations as a two-level dynamics

$$i\frac{\partial}{\partial Z}\begin{pmatrix}E_{\mathrm{i}}\\ E_{\mathrm{s}}\end{pmatrix} = \left[\frac{\mathbf{p}_{\mathrm{T}}^2}{2\bar{k}} - \begin{pmatrix}0 & \kappa^* e^{-i\Phi}\\ \kappa e^{i\Phi} & 0\end{pmatrix}\right]\begin{pmatrix}E_{\mathrm{i}}\\ E_{\mathrm{s}}\end{pmatrix}, \qquad (4)$$

Under this analogy the momentum operator is given by the transverse Laplacian operator $\mathbf{p}_{\mathrm{T}} = -i\nabla_{\mathrm{T}}$, and time is analogous to the propagation coordinate, Z. Note that since Φ depends on Z, the dynamics is "time-dependent". To eliminate this, we move to a rotating frame by making the local transformation

$$\begin{pmatrix}E_{\mathrm{i}}\\ E_{\mathrm{s}}\end{pmatrix} = U(\mathbf{r})\begin{pmatrix}\tilde{E}_{\mathrm{i}}\\ \tilde{E}_{\mathrm{s}}\end{pmatrix}, \qquad (5)$$

where

$$U(\mathbf{r}) = \begin{pmatrix} e^{-i\Phi(\mathbf{r})/2} & 0 \\ 0 & e^{i\Phi(\mathbf{r})/2} \end{pmatrix}, \qquad (6)$$

Now we multiply by $U^\dagger$ on the left and insert $\begin{pmatrix} E_\mathrm{i} \\ E_\mathrm{s} \end{pmatrix} = U \begin{pmatrix} \tilde{E}_\mathrm{i} \\ \tilde{E}_\mathrm{s} \end{pmatrix}$ to obtain

$$iU^\dagger \frac{\partial}{\partial Z}\left[U \begin{pmatrix} \tilde{E}_\mathrm{i} \\ \tilde{E}_\mathrm{s} \end{pmatrix}\right] = \left[U^\dagger \frac{\mathbf{p}_\mathrm{T}^2}{2\bar{k}} U - U^\dagger \begin{pmatrix} 0 & \kappa^* e^{-i\Phi} \\ \kappa e^{i\Phi} & 0 \end{pmatrix} U\right] \begin{pmatrix} \tilde{E}_\mathrm{i} \\ \tilde{E}_\mathrm{s} \end{pmatrix}, \qquad (7)$$

We have that

$$U^\dagger \frac{\partial}{\partial Z}\left[U \begin{pmatrix} \tilde{E}_\mathrm{i} \\ \tilde{E}_\mathrm{s} \end{pmatrix}\right] = \left(U^\dagger \frac{\partial}{\partial Z} U\right) \begin{pmatrix} \tilde{E}_\mathrm{i} \\ \tilde{E}_\mathrm{s} \end{pmatrix} + \frac{\partial}{\partial Z} \begin{pmatrix} \tilde{E}_\mathrm{i} \\ \tilde{E}_\mathrm{s} \end{pmatrix}$$

$$U^\dagger \mathbf{p}_\mathrm{T} U = \mathbf{p}_\mathrm{T} + U^\dagger [\mathbf{p}_\mathrm{T}, U] = \mathbf{p}_\mathrm{T} - iU^\dagger \nabla_\mathrm{T} U$$

$$U^\dagger \begin{pmatrix} 0 & \kappa^* e^{-i\Phi} \\ \kappa e^{i\Phi} & 0 \end{pmatrix} U = \begin{pmatrix} 0 & \kappa^* \\ \kappa & 0 \end{pmatrix}$$

Thus

$$i\frac{\partial}{\partial Z} \begin{pmatrix} \tilde{E}_\mathrm{i} \\ \tilde{E}_\mathrm{s} \end{pmatrix} = \left[\frac{\left(\mathbf{p}_\mathrm{T} - iU^\dagger \nabla_\mathrm{T} U\right)^2}{2\bar{k}} - \begin{pmatrix} 0 & \kappa^* \\ \kappa & 0 \end{pmatrix} - iU^\dagger \frac{\partial}{\partial Z} U\right] \begin{pmatrix} \tilde{E}_\mathrm{i} \\ \tilde{E}_\mathrm{s} \end{pmatrix}, \qquad (8)$$

Explicitly for our choice of U:

$$iU^\dagger \frac{\partial}{\partial Z} U = \frac{1}{2}\frac{\partial \Phi}{\partial Z} \boldsymbol{\sigma}_z$$

$$iU^\dagger \nabla_\mathrm{T} U = \frac{1}{2} \nabla_\mathrm{T} \Phi \boldsymbol{\sigma}_z$$

So, we have that:

$$i\frac{\partial}{\partial Z} \begin{pmatrix} \tilde{E}_\mathrm{i} \\ \tilde{E}_\mathrm{s} \end{pmatrix} = \left[\frac{\left(\mathbf{p}_\mathrm{T} - \frac{1}{2}\nabla_\mathrm{T}\Phi\boldsymbol{\sigma}_z\right)^2}{2\bar{k}} - \begin{pmatrix} \frac{1}{2}\frac{\partial\Phi}{\partial Z} & \kappa^* \\ \kappa & -\frac{1}{2}\frac{\partial\Phi}{\partial Z} \end{pmatrix}\right] \begin{pmatrix} \tilde{E}_\mathrm{i} \\ \tilde{E}_\mathrm{s} \end{pmatrix}, \qquad (9)$$

Renaming the terms:

$$\mathbf{M}(\mathbf{r}) = (\mathrm{Re}\,\kappa, \mathrm{Im}\,\kappa, \partial_Z\Phi/2), \qquad (10)$$

as the equivalent magnetization vector, and

$$\boldsymbol{\mathcal{A}} = \boldsymbol{\sigma}_z \nabla_\mathrm{T}\Phi/2, \qquad (11)$$

as an emergent gauge field, and

$$\begin{pmatrix} \tilde{E}_\mathrm{i} \\ \tilde{E}_\mathrm{s} \end{pmatrix} = \Psi, \qquad (12)$$

as the two-component spinor, we have

$$i\frac{\partial}{\partial Z}\Psi = \left[\frac{(\mathbf{p}_\mathrm{T} - \boldsymbol{\mathcal{A}})^2}{2\bar{k}} - \boldsymbol{\sigma}\cdot\mathbf{M}\right]\Psi, \qquad (13)$$

We now follow what is commonly done in the literature for the derivation of the magnetic topological Hall effect in the adiabatic regime, for example see[2]. It is beneficial to move to a local frame of reference, where the spin z-direction points along the magnetization. This is done by yet another local transformation:

$$\Psi = U'\Psi', \qquad (14)$$

where

$$U'(\mathbf{r}) = \exp\left(-i\frac{\theta(\mathbf{r})}{2}\boldsymbol{\sigma}\cdot\hat{\boldsymbol{\varphi}}(\mathbf{r})\right) = \cos\frac{\theta}{2}\mathbf{1} - i\sin\frac{\theta}{2}\boldsymbol{\sigma}\cdot\hat{\boldsymbol{\varphi}}, \qquad (15)$$

where $\theta$and φ are the polar and azimuthal angles and

$$\cos\theta = \frac{\mathbf{M}\cdot\hat{\mathbf{z}}}{|\mathbf{M}|}, \qquad (16)$$

$$\hat{\boldsymbol{\varphi}} = \frac{\hat{\mathbf{z}}\times\widehat{\mathbf{M}}}{\left|\hat{\mathbf{z}}\times\widehat{\mathbf{M}}\right|}, \qquad (17)$$

This transforms our equation yet again:

$$i\frac{\partial}{\partial Z}\Psi' = \left[\frac{(\mathbf{p}_\mathrm{T} - \boldsymbol{\mathcal{A}}')^2}{2\bar{k}} + V\right]\Psi', \qquad (18)$$

where

$$V = -iU'^{\dagger}\frac{\partial}{\partial Z}U' - M\boldsymbol{\sigma}_z, \qquad (19)$$

and

$$\boldsymbol{\mathcal{A}}' = iU'^{\dagger}\boldsymbol{\nabla}_\mathrm{T}U' + U'^{\dagger}\boldsymbol{\mathcal{A}}U' = iU'^{\dagger}\boldsymbol{\nabla}_\mathrm{T}U' + U'^{\dagger}\boldsymbol{\sigma}_z U'(\boldsymbol{\nabla}_\mathrm{T}\Phi/2)\,, \qquad (20)$$

are the new emergent gauge field and potential.

Supplementary Note 2: Derivation of the synthetic gauge fields

The first term in $\boldsymbol{\mathcal{A}}'$ is the same as in Ref.[2], giving rise to an emergent magnetic field which equals the skyrmion density. It is given by:

$$iU'^{\dagger}\boldsymbol{\nabla}_\mathrm{T}U' = \frac{1}{2}(\boldsymbol{\sigma}\cdot\hat{\boldsymbol{\varphi}})\boldsymbol{\nabla}_\mathrm{T}\theta - \frac{1}{2}\sin\theta\,(\boldsymbol{\sigma}\cdot\hat{\boldsymbol{\rho}})\boldsymbol{\nabla}_\mathrm{T}\varphi + \sin^2\frac{\theta}{2}\boldsymbol{\sigma}_z\boldsymbol{\nabla}_\mathrm{T}\varphi\,, \qquad (21)$$

The second term in $\boldsymbol{\mathcal{A}}'$ arises because of the original gauge field we started with. Since U' rotated the magnetization direction $\widehat{\mathbf{M}}$ to $\hat{\mathbf{z}}$, the second term in $\boldsymbol{\mathcal{A}}'$ is intuitively calculated by understanding it as a rotation of the $\hat{\mathbf{z}}$ direction in the opposite orientation ($\widehat{\mathbf{M}}$ reflected in $\theta \to -\theta$). Writing $\widehat{\mathbf{M}} = \cos\theta\,\hat{\mathbf{z}} + \sin\theta\cos\varphi\,\hat{\mathbf{x}} + \sin\theta\sin\varphi\,\hat{\mathbf{y}}$ in Cartesian coordinates, we have that

$$U'^{\dagger}\boldsymbol{\sigma}_z U'(\boldsymbol{\nabla}_\mathrm{T}\Phi/2) = \big(\cos\theta\,\boldsymbol{\sigma}_\mathbf{z} - \sin\theta\,(\boldsymbol{\sigma}\cdot\hat{\boldsymbol{\rho}})\big)(\boldsymbol{\nabla}_\mathrm{T}\Phi/2), \qquad (22)$$

So that in total

$$\mathcal{A}' = \frac{1}{2}(\boldsymbol{\sigma}\cdot\hat{\boldsymbol{\varphi}})\nabla_T\theta - \frac{1}{2}\sin\theta\,(\boldsymbol{\sigma}\cdot\hat{\boldsymbol{\rho}})\nabla_T\varphi + \sin^2\frac{\theta}{2}\boldsymbol{\sigma}_z\nabla_T\varphi + \big(\cos\theta\,\boldsymbol{\sigma}_z - \sin\theta\,(\boldsymbol{\sigma}\cdot\hat{\boldsymbol{\rho}})\big)(\nabla_T\Phi/2), \qquad (23)$$

The potential is calculated in a similar manner to the first term in $\mathcal{A}'$

$$V = -iU'^{\dagger}\frac{\partial}{\partial Z}U' - M\boldsymbol{\sigma}_z = -\frac{1}{2}(\boldsymbol{\sigma}\cdot\hat{\boldsymbol{\varphi}})\partial_Z\theta + \frac{1}{2}\sin\theta\,(\boldsymbol{\sigma}\cdot\hat{\boldsymbol{\rho}})\partial_Z\varphi - \sin^2\frac{\theta}{2}\boldsymbol{\sigma}_z\partial_Z\varphi - M\boldsymbol{\sigma}_z, \qquad (24)$$

In the adiabatic approximation, the "spin" of our light beam (its two-component color) follows the "magnetization" direction (the nonlinear coupling). Under this approximation the spin operator $\boldsymbol{\sigma}$ is locally equal to $\boldsymbol{\sigma}_z$ in the magnetization frame. Equivalently there is no crossing between the two spin eigenstates. Therefore, in the gauge potentials we may keep only the terms proportional to $\boldsymbol{\sigma}_z$, and, we also assume no dependence of $\mathbf{M}$ on Z ($\partial_Z\mathbf{M} = 0$, the equivalent of time invariance), finally giving

$$\mathcal{A}' = \boldsymbol{\sigma}_z[\sin^2\theta/2\,\nabla_T\varphi + \cos\theta\,(\nabla_T\Phi/2)], \qquad (25)$$

$$V = -M\boldsymbol{\sigma}_z, \qquad (26)$$

Since the dynamics is decoupled, we may readily omit the σ_z operator while defining an effective "charge" $q_s = \pm 1$ corresponding to each eigenvalue of σ_z, which we associate with the different spin eigenstates depending whether they point in parallel or antiparallel to the local magnetization. The synthetic magnetic field can be obtained from the vector potential

$$\mathcal{B} = \nabla_T \times \mathcal{A}' = -\frac{1}{2}\nabla_T\cos\theta \times \nabla_T\varphi + \frac{1}{2}\nabla_T\cos\theta \times \nabla_T\Phi, \qquad (27)$$

or

$$\mathcal{B} = -\frac{1}{2}\nabla_T\cos\theta \times \nabla_T(\varphi - \Phi), \qquad (28)$$

Note that for constant M (which is commonly the case considered in the literature), the term $\nabla_T\cos\theta \times \nabla_T\Phi = 0$ if M_z is time invariant, since

$$\nabla_T\cos\theta \times \nabla_T\Phi = \nabla_T\frac{M_z}{M} \times \nabla_T\int^Z 2M_z dZ' = \left(\frac{2}{M}\int^Z dZ'\right)\nabla_T M_z \times \nabla_T M_z = 0, \qquad (29)$$

Finally, this yields the synthetic magnetic field

$$\mathcal{B} = -\frac{1}{2}\nabla_T\cos\theta \times \nabla_T\varphi = -\frac{1}{2}\hat{\mathbf{z}}\big[\hat{\mathbf{M}}\cdot\big(\partial_x\hat{\mathbf{M}} \times \partial_y\hat{\mathbf{M}}\big)\big], \qquad (30)$$

and the synthetic electric field

$$\mathcal{E} = -\nabla_T V - \partial_z\mathcal{A}' = \nabla_T M - \cos\theta\,\nabla_T M_z = \frac{\nabla_T(M^2 - M_z^2)}{2M} = \frac{\nabla_T M_T^2}{2M} = \frac{M_T\nabla_T M_T}{M}, \qquad (31)$$

The Lorentz force is expressed as

$$\mathcal{F} = q_s(\mathcal{E} + \mathbf{v} \times \mathcal{B}), \qquad (32)$$

where $\mathbf{v} = \mathbf{k}_T/\bar{k}$, the beam angle with respect to the optical axis, serves as an effective velocity of the light beam in the transverse plane.

For a phase-matched interaction, $M_z = 0$ and $\boldsymbol{\mathcal{F}} = \pm\boldsymbol{\nabla}_{\mathrm{T}} M_{\mathrm{T}}$, which is the Stern-Gerlach force exerted on particles according to their spin [3,4].

Supplementary Note 3: Single skyrmion magnetization texture

Let us consider the following magnetization vector

$$\widehat{\mathbf{M}} = \sqrt{1 - m^2(\rho)}\cos(n\phi + \eta)\,\hat{\mathbf{x}} + \sqrt{1 - m^2(\rho)}\sin(n\phi + \eta)\,\hat{\mathbf{y}} + m(\rho)\hat{\mathbf{z}}, \qquad (33)$$

$$\mathbf{M} = M_0\widehat{\mathbf{M}}, \qquad (34)$$

We have that $\cos\theta = m(\rho)$ and $\varphi = n\phi$ giving the following magnetic and electric fields

$$\boldsymbol{\mathcal{B}} = -\hat{\mathbf{z}}\frac{1}{2}\frac{n}{\rho}\frac{\partial m}{\partial \rho}, \qquad (35)$$

$$\boldsymbol{\mathcal{E}} = -\hat{\boldsymbol{\rho}} M_0 m\frac{\partial m}{\partial \rho}, \qquad (36)$$

For the general skyrmion described in Supplementary Equation 33, the skyrmion number is

$$S = \frac{1}{4\pi}\int \widehat{\mathbf{M}} \cdot \left(\partial_x\widehat{\mathbf{M}} \times \partial_y\widehat{\mathbf{M}}\right) dA = \frac{n}{2}[\cos\theta]_0^{\infty}, \qquad (37)$$

For skyrmions, the magnetization in the z direction changes its sign across the domain wall, such that $S = \pm n$ is always an integer.

Neel- and Bloch-type skyrmions

For a Neel type skyrmion (antiskyrmion), $m(\rho) = \mp\cos\left(\pi\frac{\rho}{R}\right)$ and $\eta = 0$ the magnetization vector attains the following experssion

$$\mathbf{M}(\rho,\phi) = M_0\left[\sin\left(\pi\frac{\rho}{R}\right)\cos(n\phi)\,\hat{\mathbf{x}} + \sin\left(\pi\frac{\rho}{R}\right)\sin(n\phi)\,\hat{\mathbf{y}} \mp \cos\left(\pi\frac{\rho}{R}\right)\hat{\mathbf{z}}\right], \qquad (38)$$

Similarly, for a Bloch type skyrmion, $\eta = \frac{\pi}{2}$

$$\mathbf{M}(\rho,\phi) = M_0\left[-\sin\left(\pi\frac{\rho}{R}\right)\sin(n\phi)\,\hat{\mathbf{x}} + \sin\left(\pi\frac{\rho}{R}\right)\cos(n\phi)\,\hat{\mathbf{y}} \mp \cos\left(\pi\frac{\rho}{R}\right)\hat{\mathbf{z}}\right], \qquad (39)$$

in both cases $S = n$ and the magnetic and electric fields are the same

$$\boldsymbol{\mathcal{B}} = \mp\hat{\mathbf{z}}\frac{n}{2}\left(\frac{\pi}{R}\right)^2 \operatorname{sinc}\left(\pi\frac{\rho}{R}\right), \qquad (40)$$

$$\boldsymbol{\mathcal{E}} = \hat{\boldsymbol{\rho}} M_0\left(\frac{\pi}{R}\right)\cos\left(\pi\frac{\rho}{R}\right)\sin\left(\pi\frac{\rho}{R}\right), \qquad (41)$$

Example: linear domain wall

In Fig. 4 of the main text, we analyze the dynamics of THE in the presence of different domain wall distributions: the variation of the out-of-plane magnetization from one direction to the opposite. Below, we give an example of such domain wall variation, which differs from the conventional Neel- or Bloch-type skyrmion domain wall.

For a different type of domain wall, say a linear variation $m(\rho) = 1 - 2\rho/R$ as in Fig. 4b in the main text:

$$\mathbf{M}(\rho,\phi) = M\left(\sqrt{1-\left(1-\frac{2\rho}{R}\right)^2}\cos n\phi\,\hat{\mathbf{x}} + \sqrt{1-\left(1-\frac{2\rho}{R}\right)^2}\sin n\phi\,\hat{\mathbf{y}} + \left(1-\frac{2\rho}{R}\right)\hat{\mathbf{z}}\right), \quad (42)$$

The electric and magnetic fields are

$$\boldsymbol{\mathcal{B}} = -\frac{1}{2}\frac{n}{\rho}\frac{\partial}{\partial\rho}\left(\frac{R-2\rho}{R}\right) = \frac{n}{\rho R}\hat{\mathbf{z}}, \qquad (43)$$

$$\boldsymbol{\mathcal{E}} = -\hat{\boldsymbol{\rho}}M_0 m\frac{\partial m}{\partial\rho} = \hat{\boldsymbol{\rho}}\frac{2M_0}{R}\left(1-\frac{2\rho}{R}\right), \qquad (44)$$

Supplementary Note 4: Simulation results for the signal field

Below are simulation results for the THE starting with the signal frequency, instead of the idler frequency, shown in Fig. 3 of the paper. Simulation conditions are the same as described in the main text. We compare deflection from four different skyrmion numbers: $S = \pm 1, \pm 4$. The deflection is opposite to the one of the idler case, since the signal eigenstate acquires a geometric phase with the opposite sign.

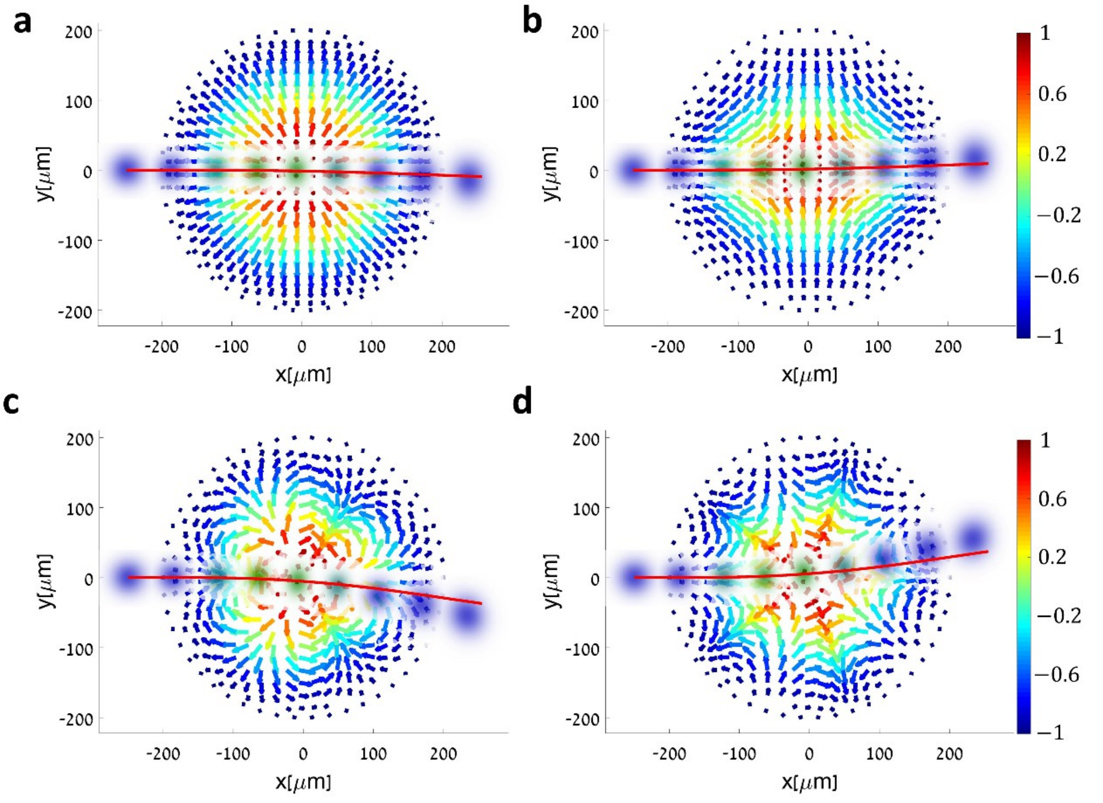

Supplementary Figure 1: **Simulation results of the topological Hall effect in skyrmionic nonlinear photonic crystals: signal frequency.** (a-b) Beam waist position on the transverse plane of the crystal, as a function of the propagation coordinate. In (a), a signal beam enters the NLPC at an angle, and traverses a Neel-type skyrmion (topological charge $S = -1$). The beam is deflected downwards, while experiencing an adiabatic change in its frequency (blue to green and back). The trajectory of a classical particle subject to the synthetic Lorentz force (Eq. 5 of the main text) is also shown (red). The colorbar reflects the z-component of the synthetic magnetization texture. In (b), the beam traverses a skyrmion with opposite topological charge $S = 1$. The beam is then deflected in the opposite direction to case (a). (c-d) Same as in (a-b), where now the light beam traverses a higher-order skyrmion ($S = \mp 4$), with a more visible difference in the deflection.

Supplementary Note 5: Topological deflection: in-plane vortex vs. skyrmion

Here we compare between the dynamics of a light beam traversing a topologically trivial in-plane vortex and a topologically nontrivial skyrmion with the same winding number. The in-plane vortex texture is given by

$$\mathbf{M}(\rho, \phi) = M_0[\cos(n\phi)\,\hat{\mathbf{x}} + \sin(n\phi)\,\hat{\mathbf{y}}], \qquad (45)$$

It is clearly seen that in the absence of topological charge in the vortex case, no detectable deflection is observed even when the winding is changed from $n = 3$ to $n = -3$. In the case of a skyrmion with a similar winding number, the topological charge $S = \pm 3$ induces a considerable difference in the deflection angle between the two cases. We avoid crossing the center, as the adiabatic approximation breaks down at the center of the vortex, so both dynamics begin with an offset with respect to the center. In the skyrmion case, the electric field attracts the idler beam towards the center (for both skyrmion numbers), thus biasing the deflection.

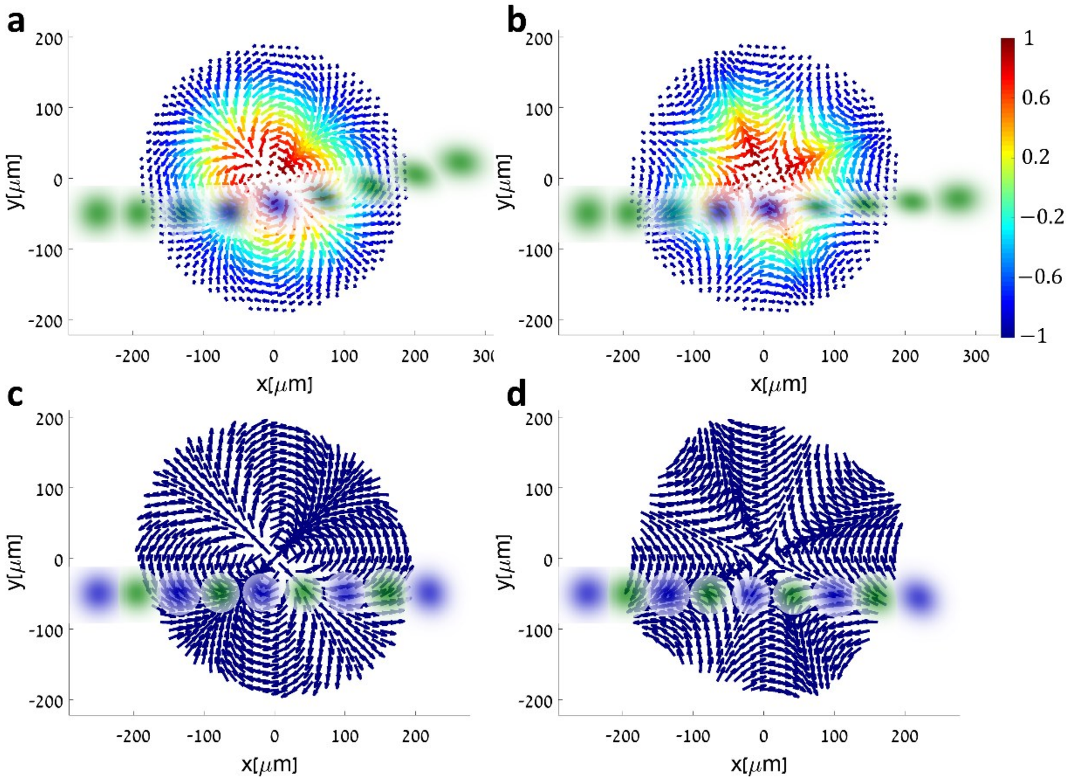

Supplementary Figure 2: **Comparison of the deflection from a skyrmion and an in-plane vortex.** (a-b) Beam waist position on the transverse plane of the crystal, as a function of the propagation coordinate. In (a), an idler beam enters the NLPC at an angle, and traverses a Neel-type skyrmion (topological charge $S = -3$). The beam is strongly deflected upwards (in part, thanks to the attractive electric field, Eq. 7a of the main text) while experiencing an adiabatic change in its frequency (green to blue and back). The colorbar reflects the z-component of the synthetic magnetization texture. In (b), the beam traverses a skyrmion with opposite topological charge $S = +3$, and the upward deflection is considerably weaker since the magnetic field has changed sign, so now its force works against the electric field. (c-d) Same as in (a-b), where now the light beam traverses an in-plane vortex with winding number $n = \pm 3$ that is topologically trivial ($S = 0$). No visible difference in the deflection is seen in this case.

Supplementary Note 6: Classical particle dynamics model

The effective dynamics of the center-of-mass motion of the beam is described by the equation

$$k\frac{d\mathbf{v}}{dZ} = q_{\mathrm{s}}(\mathbf{v}\times\boldsymbol{\mathcal{B}}+\boldsymbol{\mathcal{E}}), \qquad (46)$$

where $\mathbf{v}=\mathbf{k}_{\mathrm{T}}/k$ is a dimensionless "velocity" vector and k serves also as the effective "mass" (see main text). This equation is solved for the different eigenstates ($q_{\mathrm{s}}=\pm1$) and compared to the simulation results in Figs. 3 of the main text and Supplementary Figure 2 herein.

To obtain an approximate analytic result for the beam deflection, let us ignore the electric field, and approximate the magnetic field by a constant average value

$$\overline{\boldsymbol{\mathcal{B}}} = \frac{1}{A}\int \boldsymbol{\mathcal{B}}dA = -\frac{2\pi S}{\pi R^2} = -\frac{2S}{R^2}, \qquad (47)$$

$$\frac{d\mathbf{v}}{dZ} = \frac{q_{\mathrm{s}}}{k}\mathbf{v}\times\overline{\boldsymbol{\mathcal{B}}}, \qquad (48)$$

In the beam waist frame, the vector $\mathbf{v}$ is rotated by an angle

$$\alpha = \frac{q_{\mathrm{s}}\overline{\mathcal{B}}}{k}z_0 \cong \frac{q_{\mathrm{s}}\overline{\mathcal{B}}}{k}\frac{2R}{\tan\theta} = q_{\mathrm{s}}\overline{\mathcal{B}}\frac{2R}{k\tan\theta} = -q_{\mathrm{s}}\frac{4}{kR\tan\theta}S \cong \frac{-4q_{\mathrm{s}}}{kR|\mathbf{v}|}S, \qquad (49)$$

In total, α is linearly dependent on S, and inversely proportional to the skyrmion radius and magnitude of the velocity.

Supplementary Note 7: Nonlinear photonic crystal structure

Here we illustrate the three-dimensional structure of the nonlinear photonic crystal necessary for a skyrmion texture. The recipe for building this structure is as follows. Say that one is interested in the texture

$$\mathbf{M}(\rho,\phi) = M_0\sqrt{1-m^2(\rho)}[\cos(n\phi+\eta)\,\hat{\mathbf{x}}+\sin(n\phi+\eta)\,\hat{\mathbf{y}}] + M_0 m(\rho)\hat{\mathbf{z}}, \qquad (50)$$

Define the functions for the duty cycle, relative phase between pump and crystal and phase mismatch[5]

$$D(\rho) = \arcsin\sqrt{1-m^2(\rho)}\,/\pi, \qquad (51)$$

$$\varphi(\phi) = n\phi+\eta, \qquad (52)$$

$$\Delta k(\rho) = \Delta k_0 + 2M_0 m(\rho), \qquad (53)$$

We then build a Fourier series of the crystal structure $\mathrm{C}(\mathbf{r})$ according to

$$\mathrm{C}(\mathbf{r}) = \sum_{l=-\infty}^{\infty} c_l(\mathbf{r}), \qquad (54)$$

where the Fourier components are

$$c_0 = 2D(\rho)-1, \qquad (55)$$

$$c_{l\neq0} = \frac{2}{l\pi}\sin[l\pi D(\rho)]\exp\{il[z\Delta k(\rho)+\varphi(\phi)]\}, \qquad (56)$$

This Fourier series ensures that $\mathrm{C}(\mathbf{r}) = \pm 1$, as required by poling techniques. Practically, for the interaction only the Fourier components with $l = \pm 1$ contribute, as they are closest to phase-matching. The interaction is therefore encoded in these Fourier components such that

$$c_{+1} = \frac{2}{\pi}\sqrt{1 - m^2(\rho)}e^{i\varphi} \exp[iz\Delta k(\rho)] \propto \kappa e^{i\Phi}, \qquad (57)$$

$$c_{-1} = \frac{2}{\pi}\sqrt{1 - m^2(\rho)}e^{-i\varphi} \exp[-iz\Delta k(\rho)] \propto \kappa^* e^{-i\Phi}, \qquad (58)$$

with $\Phi = \Delta k z$, as expected.

For the simpler case of an OAM-induced skyrmion, we need only have

$$D = 1/2, \qquad (59)$$

$$\varphi = 0, \qquad (60)$$

$$\Delta k(\rho) = \Delta k_0 + 2M_0 m(\rho), \qquad (61)$$

$$c_0 = 0, \qquad c_{l\neq 0} = \frac{2}{l\pi}\sin\left(\frac{l\pi}{2}\right)\exp[ilz\Delta k(\rho)], \qquad (62)$$

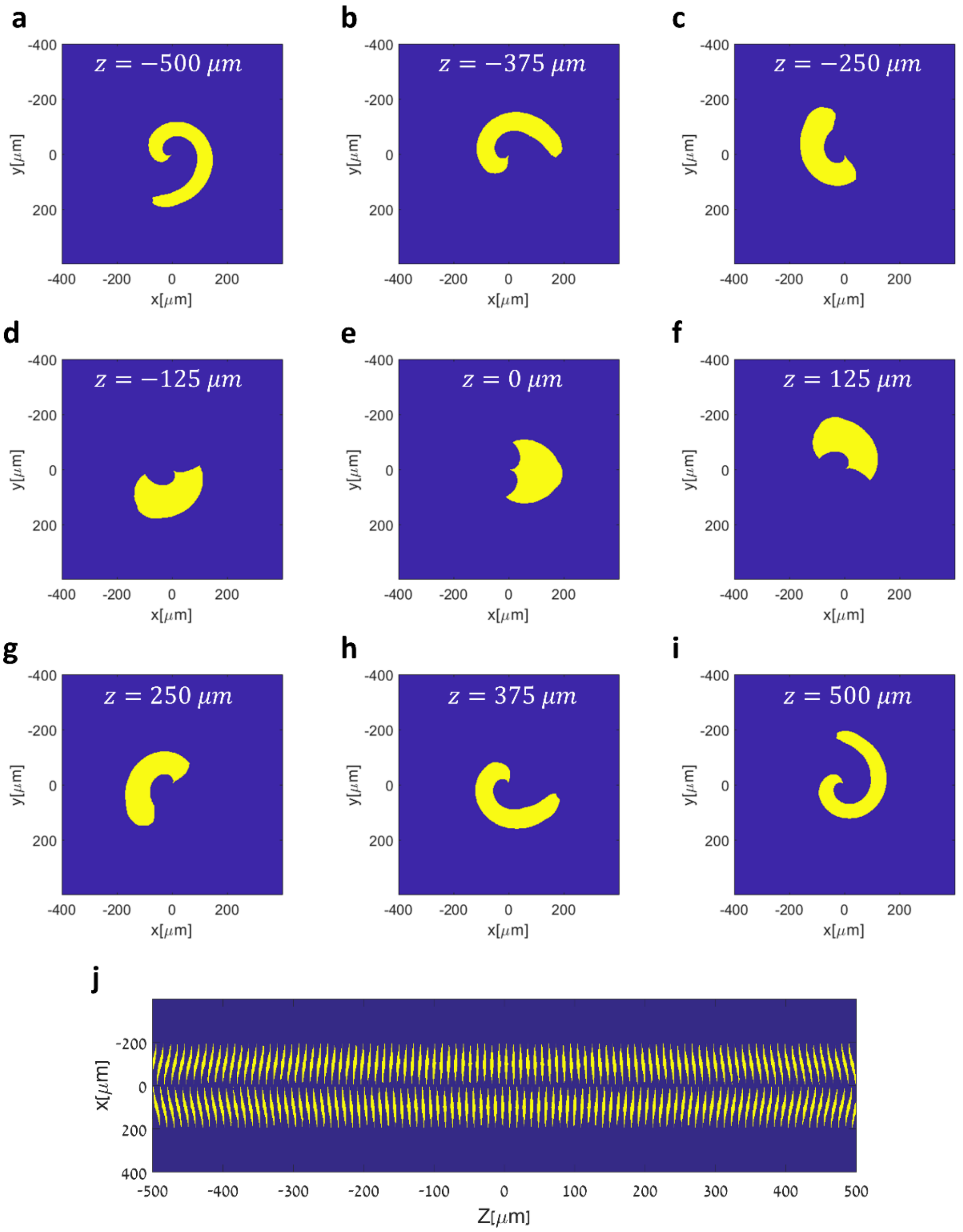

Supplementary Figure 3: **Three-dimensional nonlinear photonic crystal structure required for inducing a Neel-type skyrmion texture with skyrmion number $S = 1$.** (a-i) crystal cross-sections along the x-y plane, perpendicular to the optical axis (z), along a $1mm$ middle section of the crystal, $-500\mu m \leq z \leq 500\mu m$: (a) at $z = -500\ \mu m$ relative to the crystal center; (b) $z = -375\mu m$; (c) $z = -250\mu m$; (d) $z = -125\mu m$; (e) $z = 0\mu m$; (f) $z = 125\mu m$; (g) $z = 250\mu m$; (h) $z = 375\mu m$; and (i) $z = 500\mu m$.

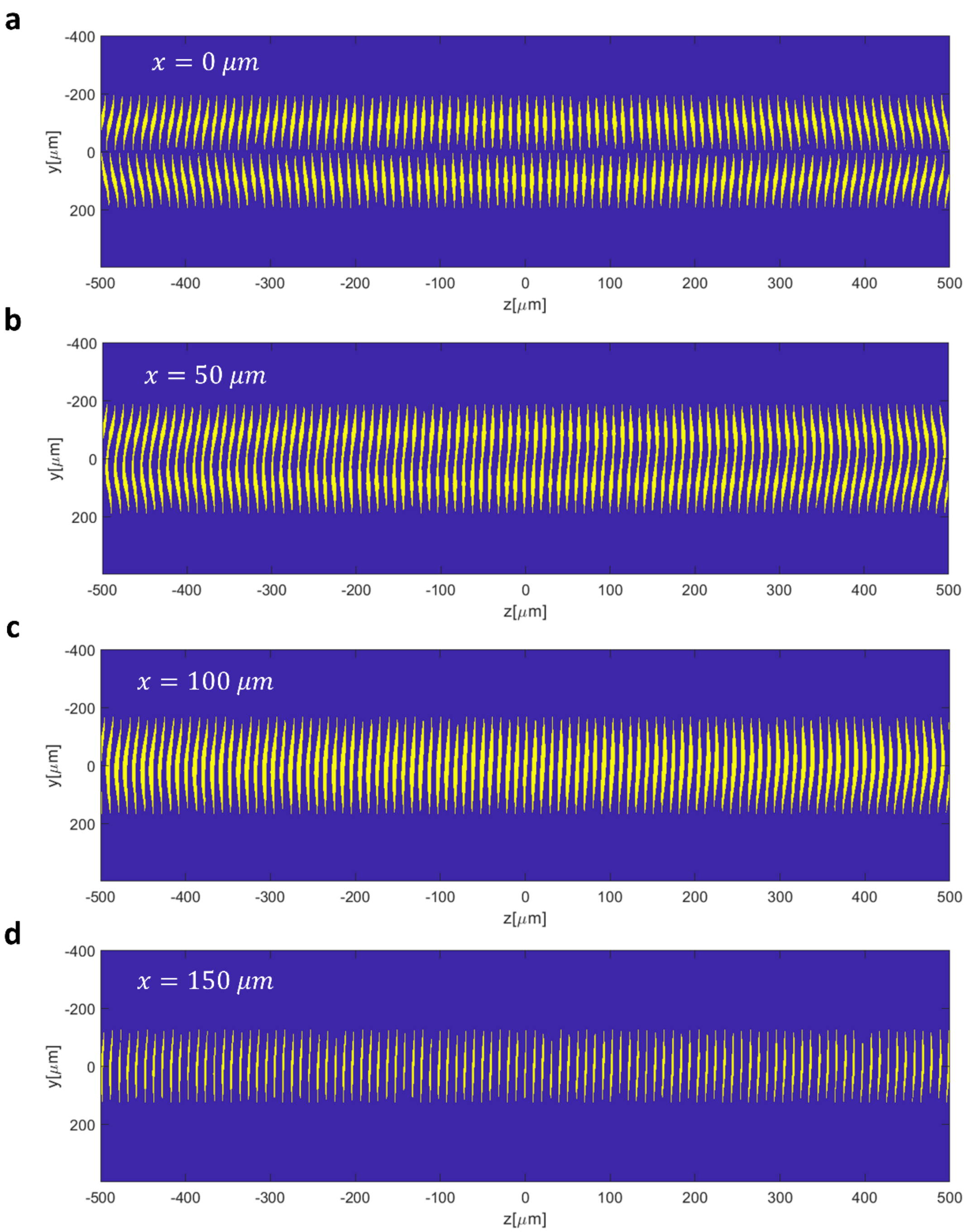

Supplementary Figure 4: **Three-dimensional nonlinear photonic crystal structure required for inducing a Neel-type skyrmion texture with skyrmion number $S = 1$.** crystal cross-section along the y-z plane, along a $1mm$ middle section of the crystal, $-500\mu m \leq z \leq 500\mu m$: (a) at $x = 0\ \mu m$ relative to the crystal center; (b) $x = 50\mu m$; (c) $x = 100\mu m$; and (d) $x = 150\mu m$.

<u>**Supplementary References**</u>

1. Boyd, R. W. *Nonlinear optics*. (Academic Press, 2008).
2. Everschor-Sitte, K. & Sitte, M. Real-space Berry phases: Skyrmion soccer (invited). *J. Appl. Phys* **115**, 172602 (2014).
3. Karnieli, A. & Arie, A. All-Optical Stern-Gerlach Effect. *Phys. Rev. Lett.* **120**, 053901 (2018).
4. Karnieli, A. & Arie, A. Frequency domain Stern–Gerlach effect for photonic qubits and qutrits. *Optica* **5**, 1297 (2018).
5. Karnieli, A. & Arie, A. Fully controllable adiabatic geometric phase in nonlinear optics. *Opt. Express* **26**, 4920 (2018).
