# Supplementary material for: Emulating spin transport with nonlinear optics, from high-order skyrmions to the topological Hall effect
Source: Nat Commun. 2021 Feb 17;12:1092. doi: 10.1038/s41467-021-21250-z (PMC7889664; doi:10.1038/s41467-021-21250-z)
Supplement: Supplementary file 2 — Description of Additional Supplementary Files [file 41467_2021_21250_MOESM2_ESM.pdf]

Description of Additional Supplementary Files

Supplementary Movie 1

Caption: Propagation of a light beam in the idler frequency inside a high-order (S = 4) skyrmionic nonlinear photonic crystal, entering at an angle from right to left with respect to the optical axis. Left: location and profile of the beam on the transverse xy plane (the z direction is the propagation axis). Beam color (green and blue) represents the adiabatic frequency conversion from the idler to the signal frequency and back as it traverses the skyrmion. Inset is the skyrmion texture (arrows indicating the direction of the synthetic magnetization), whereas color represents the value of the z component of the magnetization. Due to the synthetic Lorentz force acted on the light beam experiencing the topological Hall effect, it is deflected to the negative part of the y axis. Right: total intensity profile on the transverse plane showing the beam deflection towards negative y values.

Supplementary Movie 2

Caption: Propagation of a light beam in the idler frequency inside a high-order (S = 4) skyrmionic nonlinear photonic crystal, entering at an angle from left to right with respect to the optical axis. Left: location and profile of the beam on the transverse xy plane (the z direction is the propagation axis). Beam color (green and blue) represents the adiabatic frequency conversion from the idler to the signal frequency and back as it traverses the skyrmion. Inset is the skyrmion texture (arrows indicating the direction of the synthetic magnetization), whereas color represents the value of the z component of the magnetization. Due to the synthetic Lorentz force acted on the light beam experiencing the topological Hall effect, it is deflected to the positive part of the y axis. Right: total intensity profile on the transverse plane showing the beam deflection towards positive y values.

Supplementary Movie 3

Caption: Propagation of a light beam in the idler frequency inside a high-order (S = 2) skyrmionic nonlinear photonic crystal with a linear domain wall, entering at an angle from left to right with respect to the optical axis. Left: location and profile of the beam on the transverse xy plane (the z direction is the propagation axis). Beam color (green and blue) represents the adiabatic frequency conversion from the idler to the signal frequency and back as it traverses the skyrmion. Inset is the skyrmion texture (arrows indicating the direction of the synthetic magnetization), whereas color represents the value of the z component of the magnetization. Due to the synthetic Lorentz force acted on the light beam experiencing the topological Hall effect, it is deflected to the positive part of the y axis. Right: total intensity profile on the transverse plane showing the beam deflection towards positive y values. The linear domain wall profile enhances the Berry curvature and thus the strength of the Lorentz force.

Supplementary Movie 4

Caption: Propagation of a light beam in the idler frequency inside a high-order (S = 2) skyrmionic nonlinear photonic crystal with a cubic domain wall, entering at an angle from left to right with respect to the optical axis. Left: location and profile of the beam on the transverse xy plane (the z direction is the propagation axis). Beam color (green and blue) represents the adiabatic frequency conversion from the idler to the signal frequency and back as it traverses the skyrmion. Inset is the skyrmion texture (arrows indicating the direction of the synthetic magnetization), whereas color represents the value of the z component of the magnetization. Due to the synthetic Lorentz force acted on the light beam experiencing the topological Hall effect, it is deflected to the positive part of the y axis. Right: total intensity profile on the transverse plane showing the beam deflection towards positive y values. The cubic domain wall profile decreasesthe Berry curvature and thus the strength of the Lorentz force.
